# Karyotype analysis of eight cultivated *Allium* species

**DOI:** 10.1007/s13353-018-0474-1

**Published:** 2018-10-23

**Authors:** Farzaneh Pordel Maragheh, Daniel Janus, Magdalena Senderowicz, Kamil Haliloglu, Bozena Kolano

**Affiliations:** 1 Department of Plant Anatomy and Cytology, University of Silesia, Jagiellonska 28, 40-032 Katowice, Poland; 2 Faculty of Agriculture, Department of Field Crops, Ataturk University, 25240 Erzurum, Turkey

**Keywords:** rDNA, *Allium*, Chromosomes, FISH, CMA_3_/DAPI banding, NOR

## Abstract

The karyotypes of *Allium*, a genus that comprises many crops and ornamental plants, are relatively poorly studied. To extend our knowledge on karyotype structure of the genus, the chromosomal organization of rRNA genes and CMA/DAPI bands was studied. Fluorescence in situ hybridization using 5S and 35S rDNA probes and banding methods (silver staining and CMA_3_/DAPI staining) were used to analyze the karyotypes of eight cultivated *Allium* L. species. Analyzed *Allium* taxa revealed three different basic chromosome numbers (*x* = 7, 8, 9) and three different ploidy levels (diploid, triploid, and tetraploid). The rDNA sites chromosomal organization is reported the first time for the six species (*A*. *moly*, *A*. *oreophilum*, *A*. *karataviense*, *A*. *nigrum*, *A*. *sphaerocephalon*, *A*. *porrum*). The *Allium* species that were analyzed showed a high level of interspecies polymorphism in the number and localization of the rDNA sites. The fluorescence in situ hybridization patterns of 35S rDNA sites were more polymorphic than those of the 5S rDNA in the diploid species. Several groups of similar chromosomes could be distinguished among the chromosomes that had rDNA sites in the polyploid species. Each of the groups had three chromosomes (triploid *A*. *sphaerocephalon* L.) or four chromosomes (tetraploid *A*. *porrum* L.) suggesting their autopolyploid origin. In the genomes of four of the analyzed species, only some of the 35S rDNA sites were transcriptionally active. Fluorochrome banding revealed that the CMA_3_^+^ bands were associated with the 35S rDNA sites in all of the species that were analyzed, except *A*. *fistulosum* L. in which positive CMA_3_^+^ bands were detected in the terminal position of all of the chromosome arms. The rDNA sequences, nucleolar organizer regions (NORs), and CMA/DAPI bands are very good chromosome markers that allowed to distinguished from two to five pairs of homologous chromosomes in analyzed *Allium* species. The karyotypes of the studied species could be clearly distinguished by the number and position of the rDNA sites, NORs, and CMA/DAPI bands, which revealed high interspecific differentiation among the taxa.

**Electronic supplementary material:**

The online version of this article (10.1007/s13353-018-0474-1) contains supplementary material, which is available to authorized users.

## Introduction

The genus *Allium* L. comprises more than 800 species (Fritsch et al. 2010), thus making it one of the largest monocotyledonous genus. *Allium* consists of 15 monophyletic subgenera (Friesen et al. 2006). Species analyzed in this study belong to subgen. *Amerallium* (*A*. *moly* L.), subgen. *Allium* (*A*. *porrum* L., *A*. *sativum* L., and *A*. *sphaerocephalon* L.), subgen. *Cepa* (*A*. *fistulosum* L.), subgen. *Porphyroprason* (*A*. *oreophilum* C.A. Mey), and subgen. *Melanocrommyum* (*A*. *karataviense* Regel; Gurushidze et al. 2010; Friesen et al. 2006). *Allium* is a variable group that is spread widely across the Holarctic region from the dry subtropics to the boreal zone (Li et al. 2010; Friesen et al. 2006). Many *Allium* species are economically important plants, including, e.g., the common onion (*A*. *cepa* L.), the bunching onion (*A*. *fistulosum*), leek (*A*. *porrum*), garlic (*A*. *sativum*), and many ornamental species such as *A*. *moly* L. or *A*. *sphaerocephalon* L. (Fritsch et al. 2010). This genus exhibits a great diversity of various morphological characters, particularly in life form, (bulbs or rhizomes) and ecological habitat (Stearn 1992). *Allium* also displays a high level of diversity on the cytogenetic level: 10.64-fold differences in genome size (from 7 pg/1C in *A*. *altyncolicum* N. Friesen to 74.50 pg/1C in *A*. *validum* S. Watson; Ohri et al. 1998; Ricroch et al. 2005) and chromosome number. *Allium* has three different basic chromosome numbers *x* = 7, 8 (observed in most species) and *x* = 9 (Jones and Rees 1968). This genus, except diploids, contains many polyploid species, and the diversity in the ploidy level ranges from 2*x* to 10*x* (Bennett et al. 2000; de Sarker et al. 1997). Although the origin of most of the polyploids is not known, both allopolyploids (e.g., *A*. *sacculiferum* Maxim.) and autopolyploids (e.g., *A*. *porrum*) have been reported to date (Seo et al. 2007; Shibata and Hizume 2002; Stack and Roelofs 1996) as well as odd-ploidy plants (e.g., *Allium* × *cornutum*, which is of a triparental hybrid origin; Fredotovic et al. 2014).

Molecular cytogenetic analysis has only been performed for few species and these studies primarily focused on *A*. *cepa*, *A*. *fistulosum*, and *A*. *wakegi* Araki (a diploid hybrid between *A*. *cepa* and *A*. *fistulosum*; Shibata and Hizume 2002). The chromosomal localization of several tandem repeats and disperse repetitive sequences has been reported for these species delivering good chromosome markers for the karyotype structure and evolution analyses as well as for breeding programs (Do et al. 2001; Fajkus et al. 2016; Kirov et al. 2017; Shibata and Hizume 2002). Although in several *Allium* species the chromosomal patterns of the rDNA sites or C-banding patterns have been analyzed, most often, the karyological studies on *Allium* species have been focused on the number and morphology of chromosomes (de Sarker et al. 1997; Dolatyari et al. 2018; Murín 1964). Thus, there is a clear need to find out more about the karyotype structure in the *Allium* genus. Due to their abundance as “house-keeping genes” and their relatively conserved nature, rDNA sequences are the chromosomal markers that are most often used, especially in non-model organisms (Roa and Guerra 2015; Volkov et al. 2004). The nuclear ribosomal RNA genes encoding for 18S-5.8S-25S (35S) and 5S ribosomal RNAs (5S rDNA) consist of conserved genic regions and variably transcribed and non-transcribed spacer regions that are arranged as tandem arrays at one or more loci (Alvarez and Wendel 2003; Volkov et al. 2004). The 35S rDNA sequences are located in the nucleolar organizer regions (NORs), whereas the tandem arrays of 5S rDNA most often map independently of them (Heslop-Harrison and Schwarzacher 2011; Volkov et al. 2004). Fluorescence in situ hybridization (FISH) with 5S and 35S rDNA sequences has provided useful landmarks for chromosome identification in many plant species and has been used to construct physical maps of chromosomes as well as for phylogenetic studies in many plant species (Hasterok et al. 2006; Jang et al. 2013; Kolano et al. 2013; Roa and Guerra 2015). The mapping of ribosomal DNA through FISH is also often used as an effective tool for accurately characterizing diverse groups of germplasm materials, breeding lines, and cultivars. For example, the FISH with rDNA sequences allowed to analyze the genome re-structuring in long-term micropropagated tulips (Marasek-Ciolakowska and Podwyszynska 2008) or allowed characterization of interspecific hybrids of *Passiflora* (de Melo et al. 2017). The aim of this study was to test if the rDNA sequences and banding methods are efficient chromosome markers for karyotyping and chromosome identification in cultivated *Allium* species. FISH was used to obtain the patterns of the rRNA gene sites distribution, and silver staining was used to reveal the transcriptional activity of the 35S rDNA sites in selected cultivated *Allium* species. Additionally, double staining with CMA_3_ and DAPI was used to identify the spatial relationships between the rDNA sites and the positive CMA_3_ bands.

## Materials and methods

### Plant material and chromosome preparation

Seeds of *A*. *porrum* L. and *A*. *fistulosum* L. cv. Krolland were purchased from PlantiCo Zielonki (Stare Babice, Poland). Bulbs of *A*. *moly* L., *A*. *sphaerocephalon* L., *A*. *oreophilum* C.A. Mey., *A*. *nigrum* Sm., and *A*. *karataviense* Regel were purchased from the Benex gardening company (Chrzypsko Wielkie, Poland). Bulbs of *A*. *sativum* cv. Ornak were obtained from MARKIE-POL (Biała, Poland). Three analyzed species are well-known vegetable (*A*. *sativum*, *A*. *porrum*, and *A*. *fistulosum*). The rest of the species are frequent ornamental plant in European and North American gardens (Fritsch 2015).

*Allium* root tips 1.5–2 cm long were obtained from bulbs grown in pots in the greenhouse of Silesian University. The seeds were germinated on moist filter paper in Petri dishes. Whole seedlings (approximately 2 cm long) and the root tips that had been cut from the bulbs were pretreated with 2 mM 8-hydroxyquinoline for 3–5 h and fixed in 3:1 ethanol/acetic acid. The fixed material was washed in a 0.01 M citric acid-sodium citric buffer (pH 4.8) and digested in a mixture of 20% pectinase (Sigma P0690) and 2% cellulose (Onozuka R-10 Serva) for 1–1.5 h at 37 °C. A single root tip was washed in cold distilled water and transferred into a drop of 45% acetic acid on a microscope slide and squashed. The coverslips were removed after freezing and the slides were air-dried.

### Staining methods

Double fluorescent staining with chromomycin A3 (CMA_3_) and 4′,6-diamidino-2-phenylindole (DAPI) was used, as described by Kolano et al. (2013). The transcriptional activity of the 35S rRNA gene sites was determined using silver staining. The slides were incubated in a borate buffer (pH 9.2), air-dried, and then several drops of freshly prepared 50% (*w*/*v*) AgNO3 (Merck) in re-distilled water were applied. The slides were covered with a nylon mesh (Nylbot) and incubated in a moisture chamber for 50–70 min. at 42 °C, washed in re-distilled water, air-dried, and mounted in DPX (a mixture of distyrene, a plasticizer, and xylene; Fluka).

### Fluorescent in situ hybridization

The probe that was used to detect the 35S rRNA gene sites was a 2.3-kb fragment of the 25S rDNA coding region from *Arabidopsis thaliana* (L.) Heynh (Unfried and Grurndler 1990), which was labeled with fluorescein-12-dUTP (Roche, Switzerland). In order to detect the 5S rDNA sites, a 410-bp clone that had been isolated from *Triticum aestivum* L. (Gerlach and Dyer 1980) was amplified and labeled with dioxygenin-11-dUTP. Both DNA probes were labeled using nick translation (Roche, Switzerland).

FISH was performed according to the protocols described by Schwarzacher and Heslop-Harrison (2000). Briefly, a hybridization mixture consisting of 100 ng of a labeled DNA probe, 50% formamide, 2xSSC, 10% dextran sulfate, and 0.1% SDS was denatured for 10 min at 85 °C and then applied to the chromosome preparations. The slides and hybridization mixture were denatured together at 75 °C for 5 min in an in situ thermal cycler (Thermo Hybaid, Franklin, USA) and allowed to hybridize overnight in a humid chamber at 37 °C. Stringent washes (twice in 0.1xSSC at 42 °C) were followed by the detection of digoxigenin using the rhodamine-conjugated primary anti-digoxigenin antibody (Roche Basel, Switzerland). The signal was amplified with the Texas Red-conjugated anti-sheep secondary antibody (Jackson ImmunoResearch, Suffolk, UK). The preparations were mounted in a Vectashield antifade solution (Vector Laboratories, Peterborough, UK) containing 2 μg/ml of DAPI.

## Results

The *Allium* species that were analyzed revealed three different basic chromosome numbers *x* = 7, 8, 9. Six of the analyzed species were diploids, one species was a triploid (*A*. *sphaerocephalon*), and one species was a tetraploid (*A*. *porrum*). Studied *Allium* karyotypes mostly contained metacentric chromosomes; however, submetacentric or subtelocentric chromosomes were also observed. The karyotype formulas for each of the analyzed species are presented in Table 1.

**Table 1:** Chromosome numbers, karyotype formula, numbers of 5S and 35S rDNA sites, NORs, and CMA_3_^+^ bands in the karyotypes of analyzed *Allium* species

Species	2n	Karyotype formula	5S rDNA	35S rDNA	NOR	CMA_3_
*Allium moly*	14	2*n* = 14 = 12 m + 2sm	2	4	4	4
*A*. *oreophilum*	16	2*n* = 16 = 12 m + 2sm + 2st	4	8	4	6
*A*. *sativum*	16	2*n* = 16 = 12 m + 4sm	4	4	4	4
*A*. *fistulosum*	16	2*n =* 16 = 14 m + 2st	2	2	2	16
*A*. *karataviense*	18	2*n* = 18 = 14 m + 2sm + 2st	4	6	2	10
*A*. *nigrum*	16	2*n* = 16 = 12 m + 3sm + 1st	2	2	1	4
*A*. *sphaerocephalon*	24	2*n* = 24 = 21 m + 3sm	6	12	5	8
*A*. *porrum*	32	2*n* = 32 = 24 m + 8sm	13	8	8	8

**Fig. 1:**
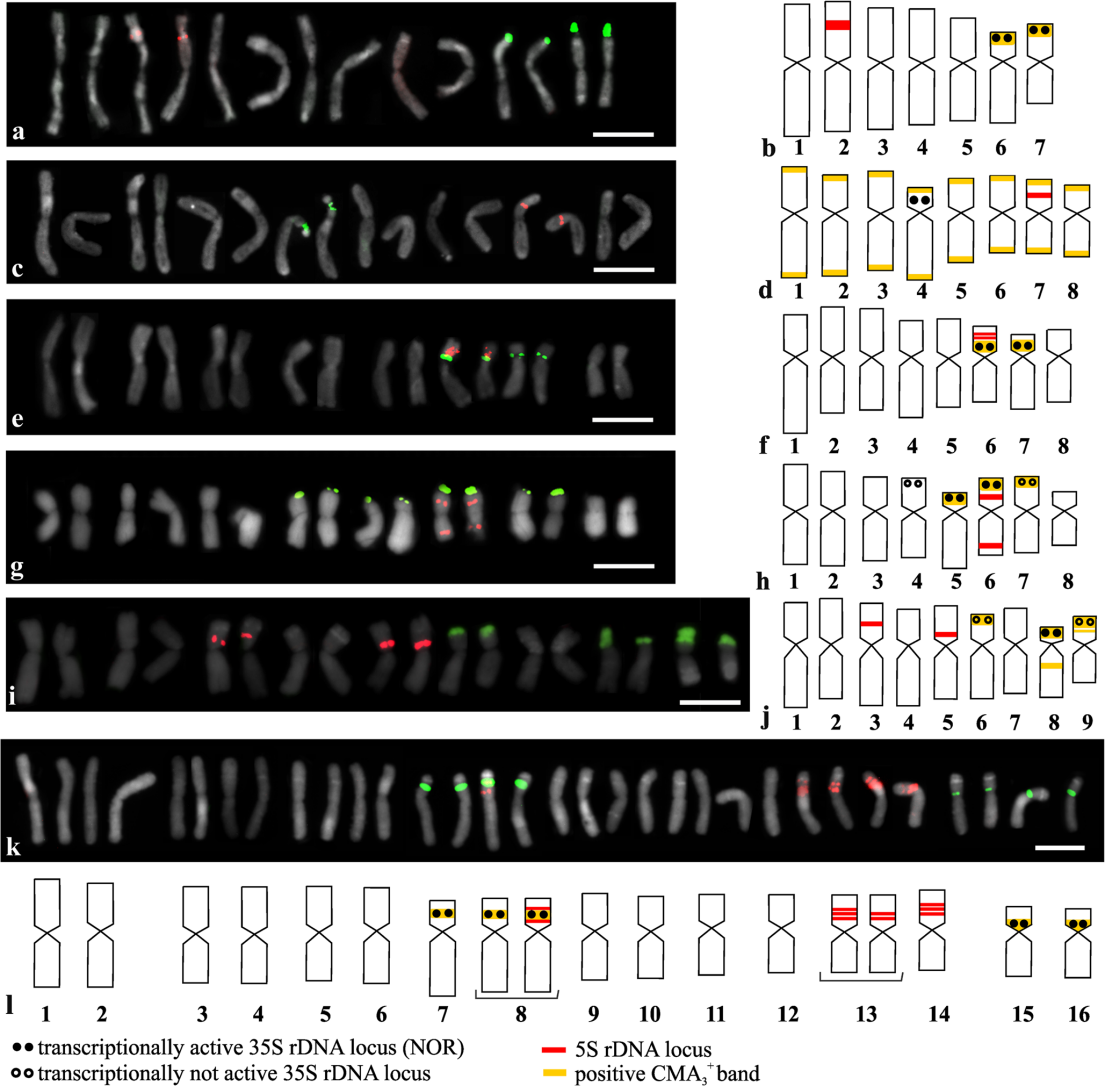
Number and localization of 35S rDNA sites (green fluorescence) and 5S rDNA sites (red fluorescence) in diploid and tetraploid *Allium* species (a, c, e, g, i, k) and idiograms of analyzed *Allium* species with localization of 35S rDNA, 5S rDNA, NORs, and positive CMA_3_ bands indicated (b, d, f, h, j, l): *A*. *moly* (a, b); *A*. *fistulosum* (c, d); *A*. *sativum* (e, f); *A*. *oreophilum* (g, h); *A*. *karataviense* (i, j); *A*. *porrum* (k, l). Only one chromosome from the homologous chromosome pair is presented in idiograms. A bracket under the chromosome in the idiograms means that there was polymorphism in the rDNA sites chromosomal organization. Bar 10 μm

The distribution of the rRNA gene sites was analyzed using FISH with 5S and 25S rDNA as probes. The analyzed *Allium* showed a high level of variability in the number and localization of the rDNA sites, and each species showed a different pattern of the rDNA sites. The 35S rDNA is expressed as a house-keeping gene with at least one pair of sites that is transcriptionally active. Silver staining was only performed for the species that had more than one pair of 35S rDNA sites. Most often, the 35S rDNA sites were colocalized with the positive CMA_3_ (CMA_3_^+^) bands. Most of the species only had negative DAPI (DAPI^−^) bands that were colocalized with the CMA_3_^+^ bands. The results of the double-target FISH to the mitotic metaphase of the *Allium* species are presented in Figs. 1, 4, and S1, and the total numbers of 5S rDNA and 35S rDNA sites are summarized in Table 1. Homologous chromosome pairs could be identified for most of the diploid species and the tetraploid *A*. *porrum*, and therefore, only one chromosome from the homologous chromosome pair is presented in the idiograms (Fig. 1).

**Fig. 2:**
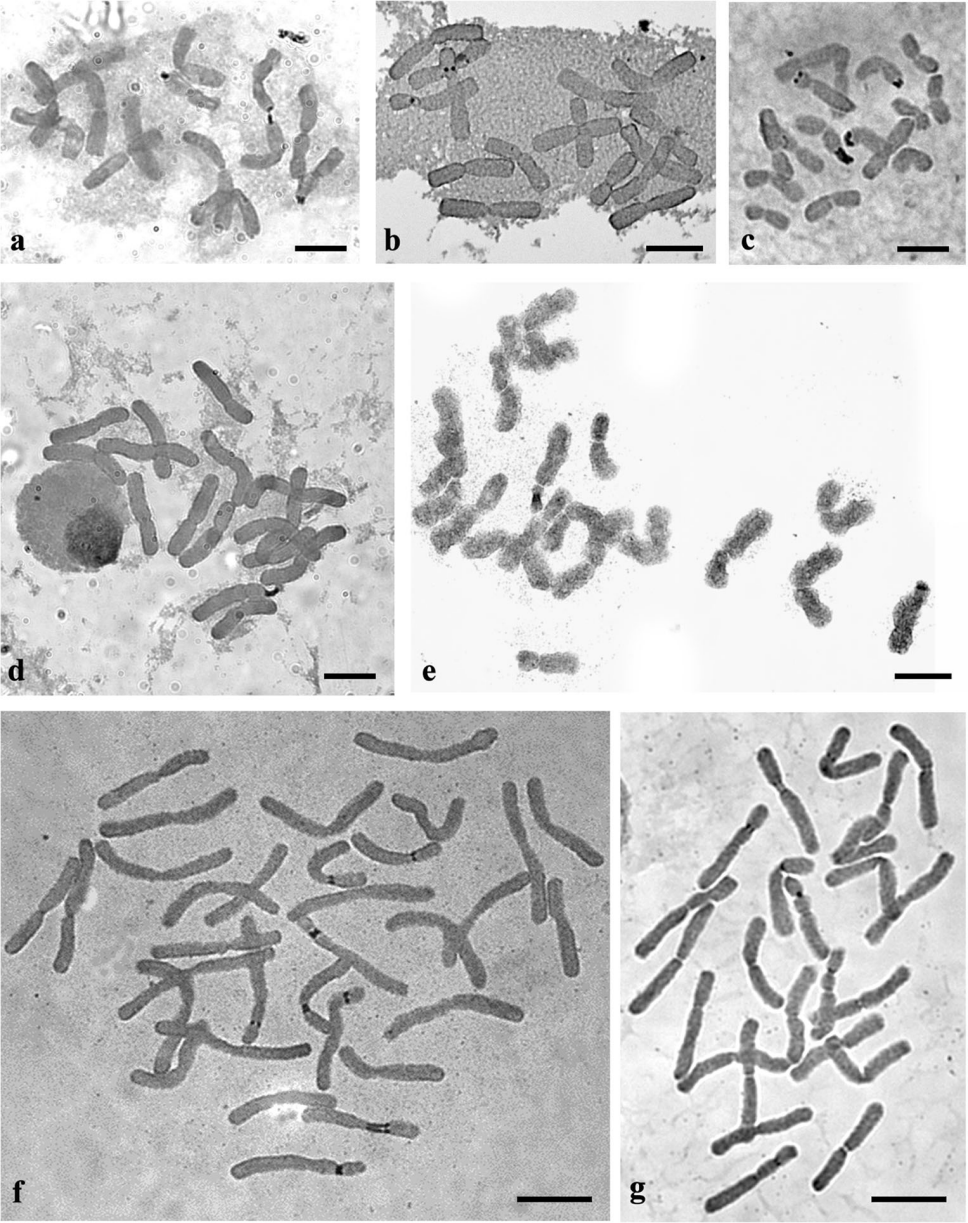
Transcriptionally active 35S rDNA sites (nucleolar organizing region; NOR) in *Allium* spp. that were detected by silver staining: *A*. *moly* (**a**); *A*. *sativum* (**b**); *A*. *oreophilum* (**c**); *A*. *nigrum* (**d**); *A*. *karataviense* (**e**); *A*. *porrum* (**f**); *A*. *sphaerocephalon* (**g**). Bar 10 μm

**Fig. 3:**
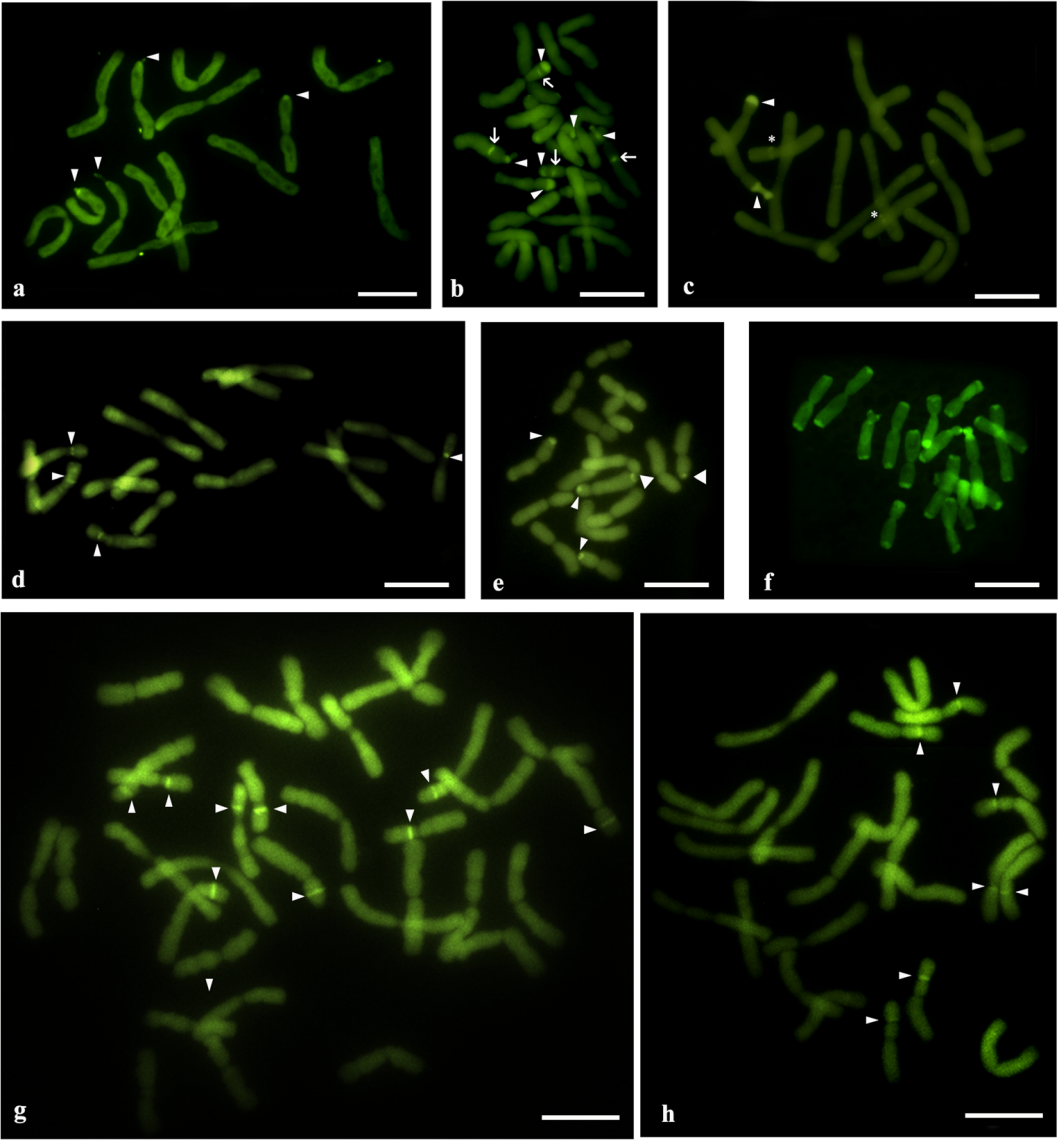
CMA_3_ fluorescent-stained chromosome complements of *Allium* species: *A*. *moly* (**a**); *A*. *karataviense* (**b**); *A*. *nigrum* (**c**); *A*. *sativum* (**d**); *A*. *oreophilum* (**e**); *A*. *fistulosum* (**f**); *A*. *porrum* (**g**); *A*. *sphaerocephalon* (**h**). The arrowheads indicate the CMA_3_^+^ bands that colocalized with the 35S rDNA sites. The arrows indicate the positive CMA_3_ bands that did not colocalize with 35S rDNA or 5S rDNA. The stars indicate the CMA_3_^+^ bands that colocalized with 5S rDNA. Bar 10 μm

The diploid *A*. *moly* (2*n* = 14) had two pairs of chromosomes (number 6 and 7) that had 35S rDNA sites in the subterminal position on the short chromosome arms. Interstitial 5S rDNA sites were observed on the short arm of one chromosome pair (number 2; Fig. 1(a, b)). All of the observed 35S rDNA sites were transcriptionally active (Fig. 1(b) and Fig. 2a) and were colocalized with the CMA_3_^+^ bands (Fig. 1(b) and Fig. 3a). The second analyzed species, diploid *A*. *fistulosum* (2*n* = 16), had only one chromosome pair with 35S rDNA sites and one chromosome pair with 5S rDNA sites. These two types of rDNA sites were localized in the interstitial position on the short arm of chromosome pair numbers 4 (35S rDNA) and 7 (5S rDNA; Fig. 1(c, d)). Neither 35S rDNA nor 5S rDNA was colocalized with the CMA_3_^+^ bands; instead, the CMA_3_^+^ bands were observed in the terminal position on each chromosome arm (Fig. 1(c) and Fig. 3f). In the garlic karyotype (*A*. *sativum*; 2*n* = 16), two pairs of 35S rDNA sites were observed in the pericentromeric position on the short arm of two chromosome pairs (numbers 6 and 7; Fig. 1(e, f)). All of the 35S rDNA sites were transcriptionally active and were colocalized with the CMA_3_^+^ bands (Fig. 1(f), Fig. 2b, and Fig. 3d)_._ Four hybridization signals of 5S rDNA were observed on the chromosome pair 7. On each of the chromosomes, two adjacent sites were present in the interstitial position on the short arm (Fig. 1(e, f)). Double FISH indicated that in the somatic cells of *A*. *oreophilum* (2*n* = 16), four pairs of 35S rDNA sites were localized in the subterminal position on the short arm of chromosome pairs 4, 5, 6, and 7 (Fig. 1(g, h)). Only half of these were transcriptionally active (chromosome pairs 5 and 6; Fig. 1(h) and Fig. 2c). Three pairs of the 35S rDNA sites (chromosome pairs 5, 6, and 7) were colocalized with the CMA_3_^+^ bands (Fig. 1(h) and Fig. 3e). Four hybridization signals of 5S rDNA were observed on chromosome pair 6. These were localized in the interstitial position on the short and long arms of the chromosomes (Fig. 1(i, h)). The next diploid species *A*. *karataviense* (2*n =* 18) had three pairs of 35S rDNA sites on the short arms of chromosome pairs 6, 8, and 9, all of which were in the subterminal position (Fig. 1(i, j)). Only one pair of 35S rDNA sites (chromosome 8) was transcriptionally active (Fig. 1(j) and Fig. 2e). All of the 35S rDNA sites colocalized with the CMA_3_^+^ bands. Additional CMA_3_^+^ bands were also observed on the long arm of chromosome pair 8 and on the short arm of chromosome pair 9, both in the interstitial position (Fig. 1(j) and Fig. 3b).

In the karyotype of the tetraploid *A*. *porrum* (2*n* = 32), it was possible to distinguish 16 pairs of homologous chromosomes, which could be further assembled into eight groups (four chromosomes in each). Four pairs of 35S rDNA sites were observed in this karyotype. Two of these were localized in the interstitial position on the short arm of chromosome pairs 7 and 8. Two others were localized in the pericentromeric position on the short arm of chromosome pairs 15 and 16 (Fig. 1(k, l)). All of the 35S rDNA sites were transcriptionally active and were colocalized with the positive CMA_3_ bands (Fig. 1(l), Fig. 2f, and Fig. 3g). Most of the 5S rDNA sites were localized in chromosome pairs 13 and 14. The pair 14 had three sites of 5S rDNA that were localized interstitially on the short arm of each chromosome. Two or three sites of 5S rDNA were observed on the chromosome 13, thus indicating polymorphisms in the number of sites between the homologous chromosomes of the same karyotype (Fig. 1(k, l)). Additionally, in the short arm of one chromosome from pair 8, two sites of 5S rDNA that flanked the 35S rDNA site were observed (Fig. 1(k, l)).

**Fig. 4:**
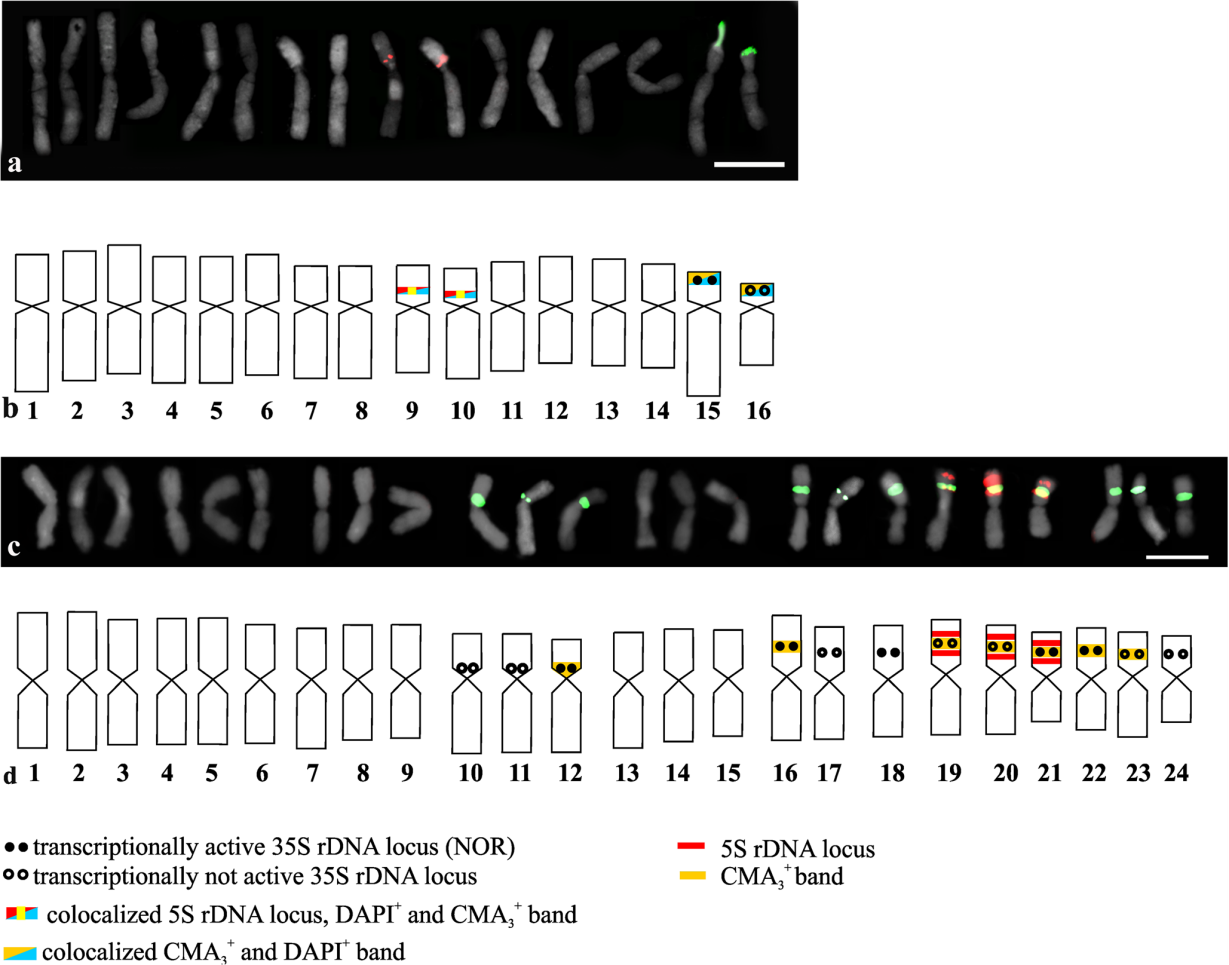
Number and localization of 35S rDNA sites (green fluorescence) and 5S rDNA sites (red fluorescence) and idiograms of *A*. *nigrum* (a, b) and the triploid *A*. *sphaerocephalon* (c, d) with the localization of 35S rDNA, 5S rDNA, NORs, positive CMA_3_ bands, and positive DAPI bands indicated. All of the chromosomes from the karyotype are presented in the idiograms. Bar 10 μm

In the karyotypes of the other two analyzed species (*A*. *nigrum* and *A*. *sphaerocephalon*), it was very difficult to identify the homologous chromosome pairs, and for these two species, all of the chromosomes are presented in the karyograms and idiograms (Fig. 4). Hybridization signals of 5S rDNA were observed on the short arms of two chromosomes (9 and 10), and two signals of 35S rDNA were observed in the interstitial position on the short arms of two other chromosomes (15 and 16) in *A*. *nigrum* (2*n* = 16; Fig. 4(a, b)). Interestingly, the chromosomes with 35S rDNA sites differed in length and morphology significantly. Moreover, the chromosomes that had 5S rDNA sites differed slightly in their morphology and in the localization of the 5S rDNA hybridization signals (Fig. 4(a, b)). Silver staining indicated that only one site of 35S rDNA in chromosome 15 was transcriptionally active (Fig. 2d and Fig. 4(b)). Two bright positive bands of CMA_3_ that colocalized with 35S rDNA sites were observed (Figs. 3c and 4b). In addition to the bright bands, a few quite dull CMA_3_^+^ bands were detected in the chromosomes. Two of these, which were observed quite consistently in the karyotype of *A*. *nigrum*, colocalized with the 5S rDNA sites (Figs. 3c and 4b). This species also had four positive DAPI bands. Two of these were colocalized with the 35S rDNA sites, and the remaining DAPI^+^ bands were colocalized with the 5S rDNA sites (Fig. 4(b) and Fig. S2c).

In the triploid *A*. *sphaerocephalon* (2*n* = 24), nine interstitially localized signals of 35S rDNA were detected on the short arms of nine metacentric chromosomes (chromosomes number 16–24). Three other 35S rDNA sites were localized in the pericentromeric position on the short arms of chromosomes 10, 11, and 12 (Fig. 4(c, d)). Silver staining indicated that only five sites were transcriptionally active—one pericentromeric site on chromosome 12 and four interstitial sites in chromosomes 17, 18, 21, and 22 (Fig. 4(d) and Fig. 2g). Eight positive CMA_3_ bands were observed in *A*. *sphaerocephalon*. All of these were colocalized with the 35S rDNA sites (Fig. 4(d) and Fig. 3h). Hybridization signals of 5S rDNA were observed on three chromosomes, and each of the chromosomes had two sites of the 5S rRNA genes that flanked the site of 35S rDNA (Fig. 4(c, d)).

## Discussion

The analyzed *Allium* species represent three different basic chromosome numbers (*x* = 7, 8, and 9) that had previously been described for this genus (Jones and Rees 1968). The somatic numbers of chromosomes 2*n* = 14, 16, and 18 of the analyzed diploid species are mainly in accordance with those that are available in the index to plant chromosome number (www.tropicos.org/Project/IPCN). In the karyograms of most of the analyzed diploids, it was possible to distinguish pairs of homologous chromosomes except for *A*. *nigrum*. In the karyotype of *A. nigrum*, the chromosomes cannot be unambiguously arranged in homologous pairs. This was especially apparent in the case of the two chromosomes that had 35S rDNA sites and might suggest a hybrid origin of this accession. Except diploid species, the *Allium* genus also contains many polyploids, which are mostly tetraploid (such as the analyzed *A*. *porrum*) and hexaploids (e.g., *A*. *parodi*; Pastor 1982). Odd-ploidy polyploids were also observed (e.g., the analyzed *A*. *sphaerocephalon* or another triploid *Allium* × *cornutum*; Fredotovic et al. 2014). *A*. *porrum* and *A*. *sphaerocephalon* were suggested to be autopolyploids based on their karyotype structure and synaptic behavior (Loidl and Jones 2004; Stack and Roelofs 1996). The chromosomal organization of the rDNA sites that were observed in these species supports this hypothesis. Based on the chromosomal patterns of the rDNA sites, it was possible to distinguish groups of chromosomes (three groups with four chromosomes in *A*. *porrum* and four groups with three chromosomes each in *A*. *sphaerocephalon*; Fig. 1(l) and Fig. 4(d)) with very similar rDNA site patterns, which could support their autopolyploid origin. On the other hand, the chromosomes that had a similar pattern of rDNA sites showed significant differences in length at least in the *A*. *sphaerocephalon* karyotype that could suggest either an allopolyploid origin or a major reorganization of the chromosomes after polyploidization. Although earlier reports showed that *A*. *sphaerocephalon* is a complex species with diploid, triploid, and tetraploid cytotypes, the origin of polyploid cytotypes was not studied (Johnson and Ozhatay 1996). Further research using molecular phylogenetic methods and cytogenetic (GISH) is necessary to elucidate the origin of the polyploids.

The present report gives the first description of the rDNA localization for six species: *A*. *moly*, *A*. *oreophilum*, *A*. *karataviense*, *A*. *nigrum*, *A*. *sphaerocephalon*, and *A*. *porrum*. The number and localization of the rDNA sites that were obtained for *A*. *fistulosum* consents with most of the earlier reports (Kirov et al. 2017; Lee et al. 1999; Son et al. 2012). However, Gernand et al. (2007) reported additional one or four minor polymorphic sites of 35S rDNA on *A*. *fistulosum* chromosomes. Only two pairs of 5S rDNA sites were observed on the short arm of one chromosome pair in *A*. *sativum*, although earlier reports showed three pairs of sites of 5S rDNA in this species (one on the long arm and two on the short arm of the same chromosome pair; Lee et al. 1999; Son et al. 2012). Such phenomenon, the intraspecific polymorphisms in the rDNA sites number, has also been observed in many species including *Amaranthus*, *Chenopodium*, and *Prospero* (Jang et al. 2013; Kolano et al. 2013; Kolano et al. 2015).

The median plant karyotype has one or two pairs of interstitial 5S rDNA sites and two pairs of subterminal 35S rDNA sites (Garcia et al. 2017; Roa and Guerra 2012; Vitales et al. 2017). The studied *Allium* species most often had more than two pairs of 5S rDNA, mostly in the interstitial and/or pericentromeric position (present study; Vitales et al. 2017; http://www.plantrdnadatabase.com). In *Allium*, the 35S rDNA sites were observed most often in the subtelomeric position on the short arm similar to many other angiosperms, and the number of 35S rDNA sites ranges from one pair to four pairs in diploid species (Roa and Guerra 2012). In polyploid species, up to 12 hybridization signals of 35S rDNA were observed in the somatic cells (triploid *A*. *sphaerocephalon*). However, it must be noted that to date, the rDNA sites organization has only been analyzed in less than 30 *Allium* species, and therefore, it is difficult to make any general conclusion on the rDNA sites distribution in the entire genus.

The chromosomal patterns of both 35S rDNA and 5S rDNA site distribution appear to be quite variable in the *Allium* genus (these data and earlier reports; http://www.plantrdnadatabase.com/). This phenomenon has been reported in many different plant genera including *Brassica* and *Paphiopedilum* (Hasterok et al. 2006; Lan and Albert 2011). Similar to many other species, the FISH patterns of the 35S rDNA sites in the *Allium* species were more polymorphic than those of the 5S rDNA (Chiarini et al. 2017; Garcia et al. 2017; Jang et al. 2016). In addition, an intraspecific polymorphism was observed in the 5S rDNA sites number in *A*. *porrum* where differences in site distributions were observed between the homologous chromosomes of one karyogram. A numerical variation in the rDNA sites has been observed in several plant species, both cultivated (e.g., *Brassica rapa*, *Amaranthus caudatus*; Hasterok et al. 2006; Kolano et al. 2013) and wild species (e.g., *Prospero autumnale*; Jang et al. 2013). However, the strong conservation of rDNA site number has been described in many plant species or even in entire genera, e.g., *Glycine* and *Daucus* (Iovene et al. 2008; Singh et al. 2001). The interspecies and intraspecific variation in the number and localization of rDNA sites has been attributed to various mechanisms such as transposon-mediated transposition events, a homologous and/or non-homologous unequal crossing over, and gene conversion and chromosomal rearrangements, such as locus duplication/deletion (Raskina et al. 2008; Altinkut et al. 2006; Datson and Murray 2006; Thomas et al. 1996), but the current data do not permit more detailed inferences of these mechanisms in *Allium*.

Double fluorescent staining with chromomycin A_3_ (CMA_3_) and DAPI was used to localize the chromosome regions that are rich in GC and AT base pairs, respectively (Schweizer 1976). In most of the analyzed *Allium* species, the regions that were occupied by the 35S rRNA genes were the only large GC-rich blocks of chromatin as was shown earlier for many plants (Guerra 2000). In two species (*A*. *oreophilum* and *A*. *sphaerocephalon*), the number of CMA_3_^+^ bands was smaller than the number of 35S rDNA sites. Whereas, three other species *A*. *karataviense*, *A*. *nigrum*, and *A*. *fistulosum* had more CMA_3_ bands than the number of 35S rDNA sites. In *A*. *nigrum*, two additional CMA_3_^+^ bands corresponded to the 5S rDNA sites were observed. There is little data on the occurrence of CG-rich heterochromatin with 5S rRNA genes (Cabral et al. 2006; Hamon et al. 2009; Kolano et al. 2013). Such an observation could reflect both the composition of the 5S rDNA sequences and the nature of the adjacent heterochromatin. *A*. *karataviense* had only two additional CMA3^+^ bands (that did not colocalize with 35S rDNA or 5S rDNA), while the patterns of the CMA_3_ bands that were observed in *A*. *fistulosum* diverged from the most frequently reported patterns. The distribution of the CMA_3_^+^ bands that were observed in *A*. *fistulosum* resembles the one that was reported in *A*. *cepa* (Kim et al. 2002). The CMA_3_^+^ bands could also correspond to the heterochromatin bands, which are mainly composed of satellite repeats (Chiarini et al. 2014; da Costa Silva et al. 2014; Do et al. 2001). The CMA_3_^+^ bands, observed in the terminal position of each chromosome arm of *A*. *cepa* and *A*. *fistulosum*, appeared to colocalize with the terminal heterochromatin that has been observed in these closely related species (Do et al. 2001; Fesenko et al. 2002; Kirov et al. 2017). Positive DAPI bands were only observed in *A*. *nigrum*, thus suggesting the presence of heterochromatin blocks containing AT-rich repetitive sequences. These bands appeared to be colocalized with positive CMA_3_ and 35S or 5S rDNA sites. DAPI^+^/CMA^+^ bands have rarely been described in plants; however, they were reported for *Cestrum* (Fernandes et al. 2009). It is also possible that the CMA^+^ and DAPI^+^ bands actually did not colocalize but are localized very close on the chromosomes. The relatively low resolution of observation using highly condensed mitotic chromosomes did not allow these two different chromatin bands to be distinguished.

The number of rRNA genes is largely redundant in relation to what is required to sustain a ribosome assemblage; hence, only a small fraction of the rDNA units is transcribed, and significant portions of 35S rDNA sites are heterochromatinized in most eukaryotes (Volkov et al. 2004). In the diploid *Allium*, only one or two pairs of 35S rDNA sites were transcriptionally active depending on the species, while in the tetraploid *A*. *porrum*, all four pairs of 35S rDNA sites were transcriptionally active. Interestingly, *A*. *nigrum* (2*n* = 16) had only one chromosome with NOR in its diploid chromosome complement. The second site of 35S rDNA appeared to be transcriptionally inactive. Moreover, in the karyotype of the triploid *A*. *sphaerocephalon*, only one third of the 35S rDNA sites were transcriptionally active. This species has four groups of chromosomes with 35S rDNA sites (each group consists of three chromosomes), and only one chromosome in each group had NOR. The silencing of rDNA sites has been described in many hybrids and allopolyploids (nucleolar dominance), but it has also been observed in diploids with more than one pair of sites (Pikaard 2000; Tucker et al. 2010; Kolano et al. 2012). This rRNA gene silencing process involves changes in DNA methylation and histone modifications. Consequently, the epigenetic regulation of NOR sites following hybridization and/or polyploidization may vary between the parental subgenomes of hybrids or allopolyploids with a tendency toward a nucleolar dominance by one parental homolog (Borowska-Zuchowska and Hasterok 2017; Tucker et al. 2010).

In conclusion, cytogenetic studies are very helpful in phylogenetic analyses and contribute to the knowledge of the structure and evolution of genomes, which is essential in modern breeding programs. The present report shows that rDNA sequences are very good chromosome markers in *Allium*. The high variability of chromosomal patterns of rDNA sites that was observed indicates that FISH with rDNA sequences could be a very good tool for comparative analyses of *Allium* karyotypes. The very rich patterns of hybridization signals and various bands could also be very useful in studies on the origin and evolution of hybrid and polyploid species/cytotypes. This report describes the results of comparative analyses of rDNA chromosomal organization in important vegetable crops (*A*. *sativum*, *A*. *fistulosum*, and A. *porrum*) and five ornamental *Allium* species; however, in order to gain a full understanding of rDNA site organization and evolution in this large genus, a wider sampling (especially of wild species that could be used as wild genetic resources) is necessary.

## Electronic supplementary material

ESM 1 (PNG 1328 kb)

High resolution image (TIF 21994 kb)

ESM 2 (PNG 1041 kb)

High resolution image (TIF 7273 kb)
