# Supplementary figures and images for: Karyotype analysis of eight cultivated Allium species
Source: J Appl Genet. 2018 Oct 23;60(1):1–11. doi: 10.1007/s13353-018-0474-1 (PMC6373409; doi:10.1007/s13353-018-0474-1)

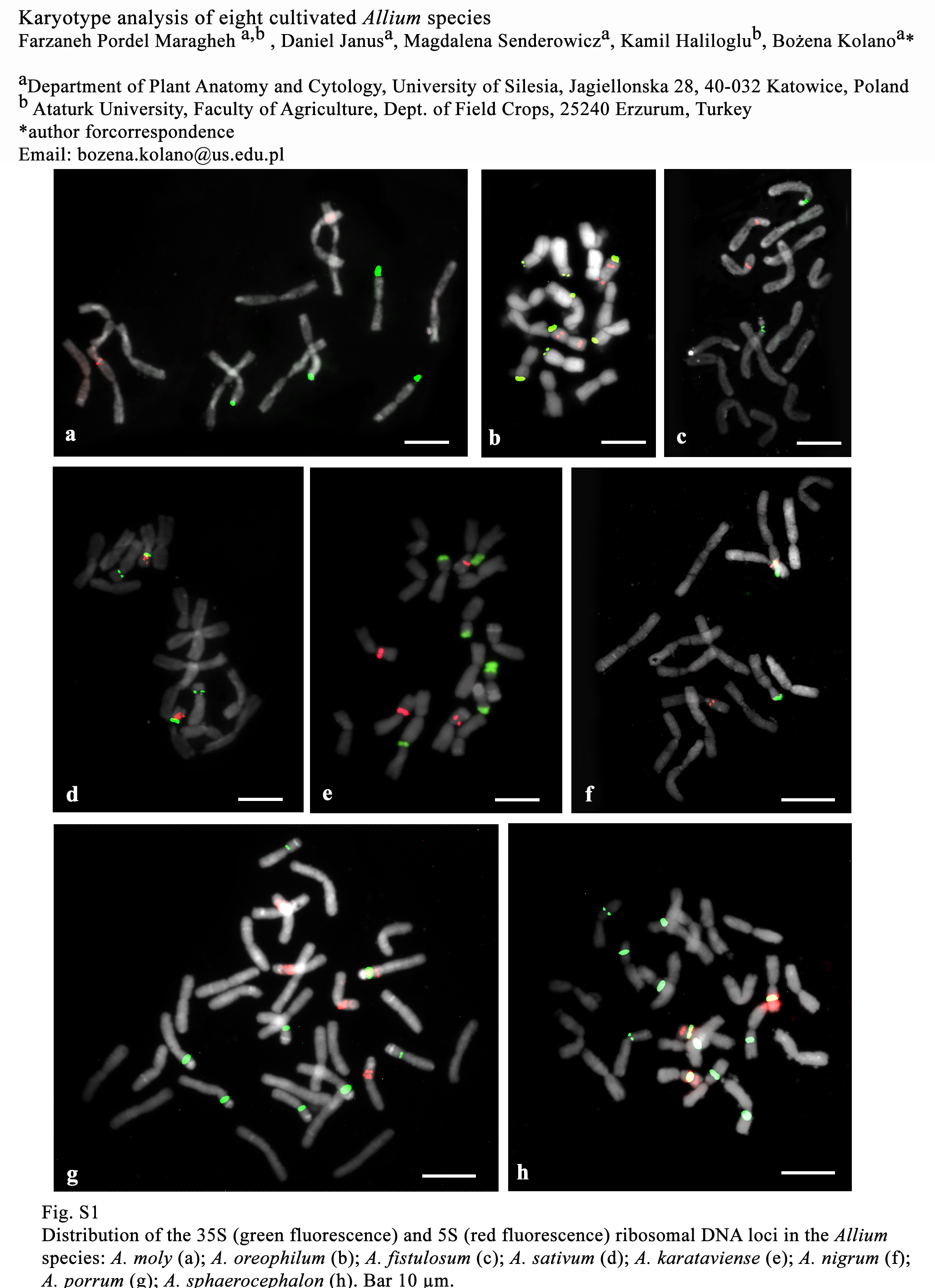

Supplement: Supplementary file 1 — (PNG 1328 kb) [file 13353_2018_474_Fig5_ESM.png]

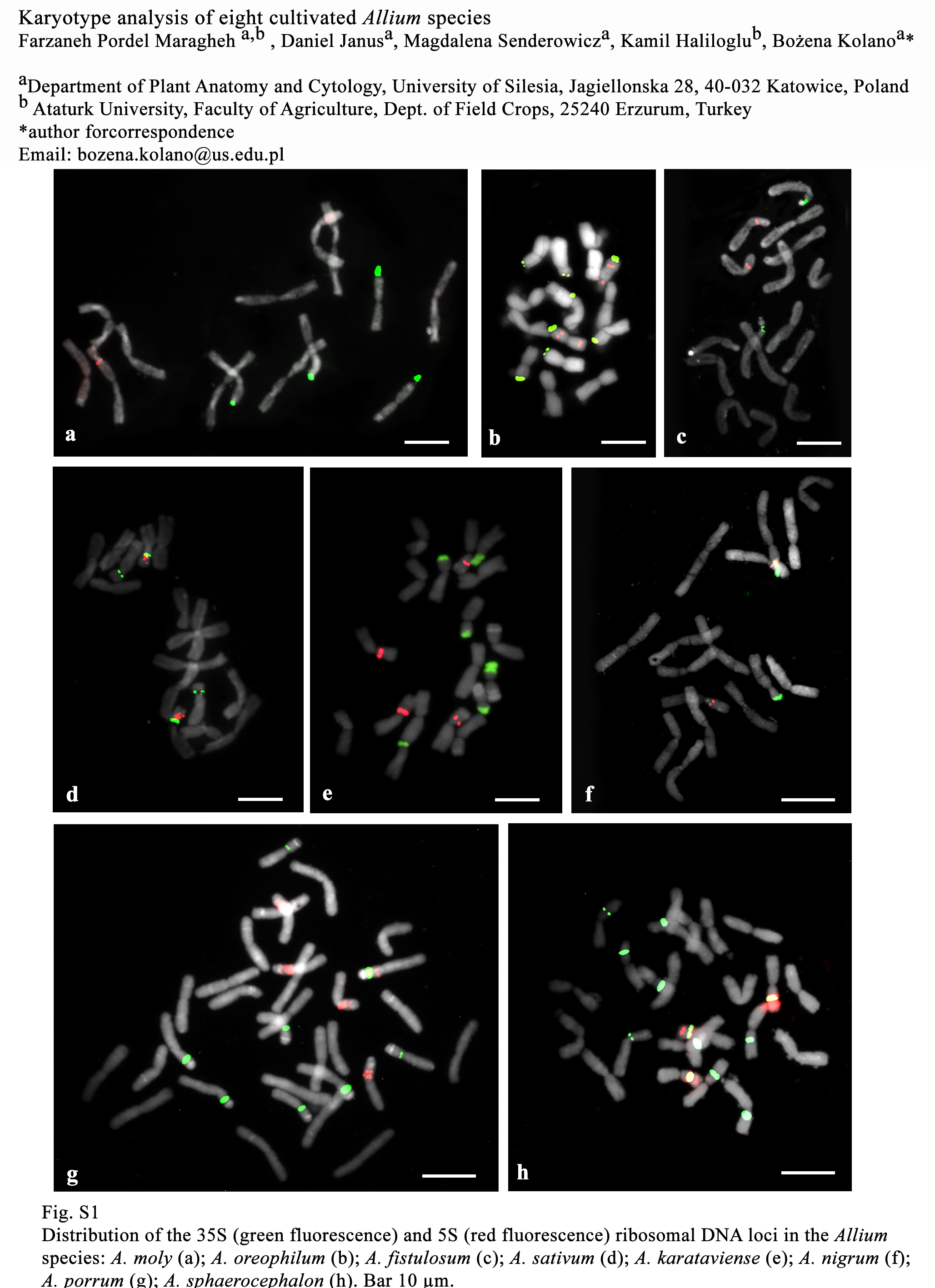

Supplement: Supplementary file 2 — High resolution image (TIF 21994 kb) [file 13353_2018_474_MOESM1_ESM.tif]

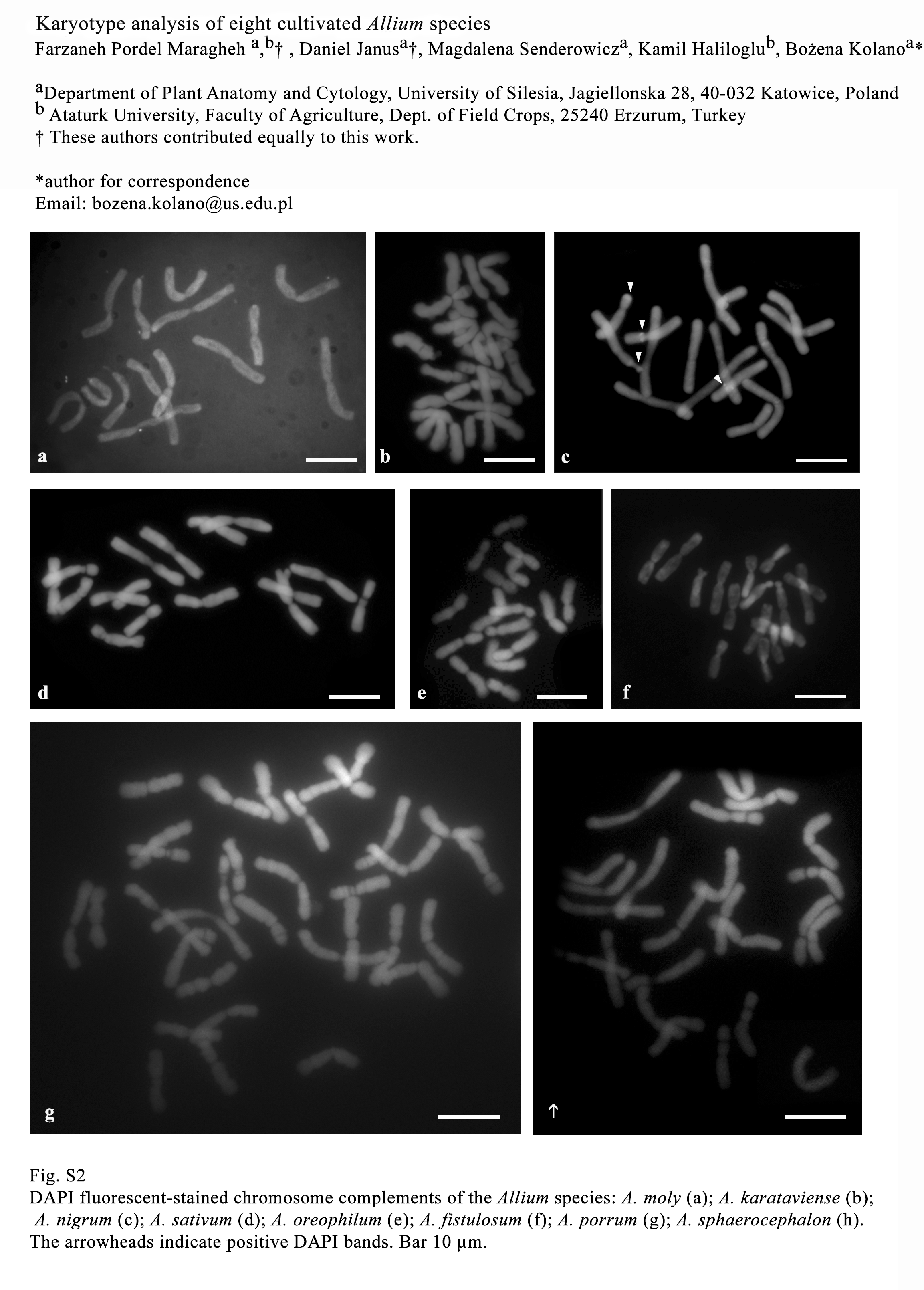

Supplement: Supplementary file 3 — (PNG 1041 kb) [file 13353_2018_474_Fig6_ESM.png]

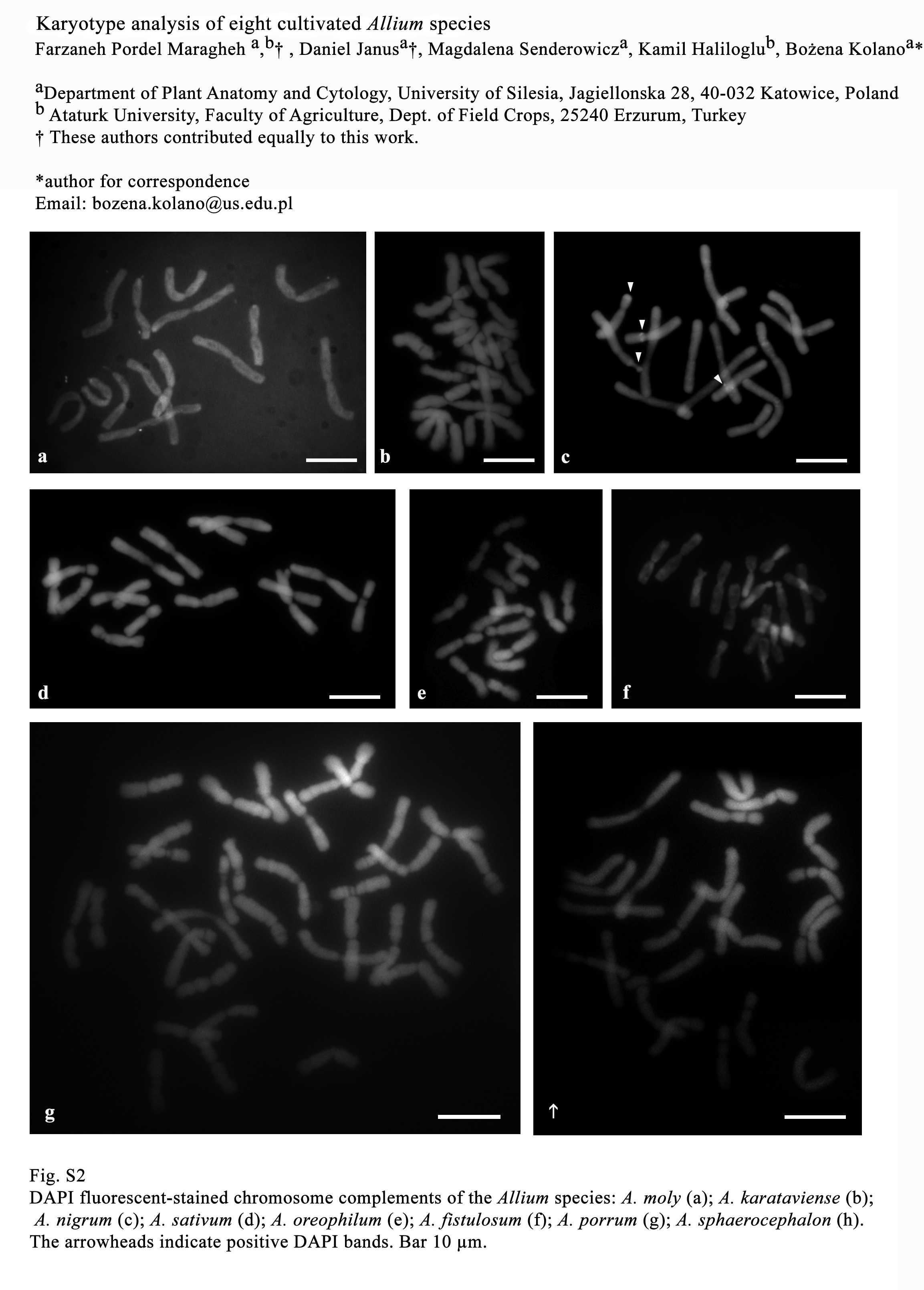

Supplement: Supplementary file 4 — High resolution image (TIF 7273 kb) [file 13353_2018_474_MOESM2_ESM.tif]
